# Multiple cholesterol granulomas of the breast: A case report and review of the literature

**DOI:** 10.1097/MD.0000000000033084

**Published:** 2023-02-22

**Authors:** Menglei Jin, Qiang Wu, Bingliang Miao, Jing Jin, Chenyi Gao, Xiaoming Xu, Yingbo Luo, Zhigang Chen

**Affiliations:** a Department of Breast Surgery, Second Affiliated Hospital, Zhejiang University School of Medicine, Hangzhou, China; b Department of Surgery, Pangang Xichang Hospital, Xichang, China; c Department of Breast Surgery, Affiliated Hospital of Shaoxing University, Shaoxing, China; d Department of Obstetrics and Gynecology, Shaoxing Maternity and Childcare Hospital, Shaoxing, China; e Department of Pathology, Second Affiliated Hospital, Zhejiang University School of Medicine, Hangzhou, China.

**Keywords:** breast, cancer, case report, cholesterol granuloma

## Abstract

**Rationale:**

Cholesterol granuloma of the breast is a rare disease defined as chronic reactive inflammation of cholesterol crystals and foreign body giant cells. This disease can mimic breast cancer in the clinic as painless palpable hard nodules, and imaging shows irregular hypoechoic nodules with unclear boundaries. Therefore, the uncommon lesions can be easily misdiagnosed as breast cancer. Meanwhile, it can be easily neglected by clinicians because of poor understanding.

**Patient concerns:**

In this report, we present a rare case of multiple cholesterol granulomas of the breast, which was analyzed retrospectively and combined with all 11 relevant available studies in the last 50 years.

**Interventions:**

The patient had undergone multiple breast imaging inspections for breast nodules and had the local resection of nodules.

**Diagnoses:**

The patient was confirmed to have a final diagnosis of benign cholesterol granulomas but was initially considered as breast cancer.

**Outcomes:**

The patient did not complain of discomfort after surgery, and ultrasound reexamination 5 months after surgery showed no recurrence.

**Lessons:**

By retrospective analysis, dynamic contrast-enhanced magnetic resonance imaging and core needle biopsy can synergistically help clinicians distinguish it from other breast disease. To raise awareness of such a rare disease and reduce related misdiagnoses, we summarize the characteristics of cholesterol granulomas and recommend appropriate novel diagnosis and treatment regimens for patients with cholesterol granulomas.

## 1. Introduction

Cholesterol granuloma is a specific form of chronic reactive inflammation with diverse causes.^[1]^ It is defined by the histopathologic pattern of cholesterol crystals and foreign body giant cells. Cholesterol granulomas are most commonly found in the middle ear and mastoid process^[2]^ but are rare in the breast. This disease was first mentioned in the mammary gland entity in 1974.^[3]^ Cholesterol granuloma of the breast is a rare disease with an estimated prevalence ranging from 54 cases per 100,00 people at the time of core needle biopsy, which occurs primarily in middle aged women.^[4]^

Although cholesterol granuloma of the breast is benign, it is confusable for clinicians to distinguish it from breast cancer simply based on clinical manifestations and imaging, which are quite similar. Some patients with cholesterol granuloma of the breast have no symptoms or discomfort. Some may present with painless palpable hard nodules. Prominent reactive fibrosis, showing hypoechoic and irregular borders at the unclear edge of the mass, indicates a radiographic mimic of cancer. Meanwhile, patients past personal history of carcinoma^[4]^ may reinforce the suspicion of malignancy. Therefore, considering the fact that there may be excessive treatment and misdiagnosis, some recent advanced imaging and biopsy techniques have played a crucial role in its diagnosis. Conservative observation can be appropriate when the pathological diagnosis of cholesterol granuloma is definitely confirmed.

Based on imaging results and pathological characteristics, we retrospectively aimed to propose available diagnostic and therapeutic approaches for this rare benign disease to deepen the understanding and reduce the possibility of misdiagnosis. This report was written following the 2020 Surgical Case Report guidelines.^[5]^ Written informed consent was obtained from the patient for publication of this case report. And this retrospective study was reviewed and granted approval by the Ethics Committee of the Second Affiliated Hospital, Zhejiang University School of Medicine.

## 2. Case report

A 61-year-old woman presented with nodules in both breasts for 2 years and was admitted to the Department of Breast Surgery of the Second Affiliated Hospital of Zhejiang University School of Medicine in April 2021. She was found to have bilateral palpable breast nodules on physical examination without tenderness, nipple bleeding or skin ulceration 2 years prior and was advised to follow up. The patient had a history of hypertension for more than 10 years and diabetes for more than 3 years. She had no history of breast trauma surgery and no family history of malignant tumors. After regular observation, the changes of the masses were not obvious. Superficial lymph nodes in the upper part of the neck and clavicle were not swollen. The breasts were symmetrical, and the skin of the breasts was not red and swollen. The masses were hard and mobile. There was no adhesion to the chest wall or skin. No enlarged axillary lymph nodes were palpated.

**Figure 1.**
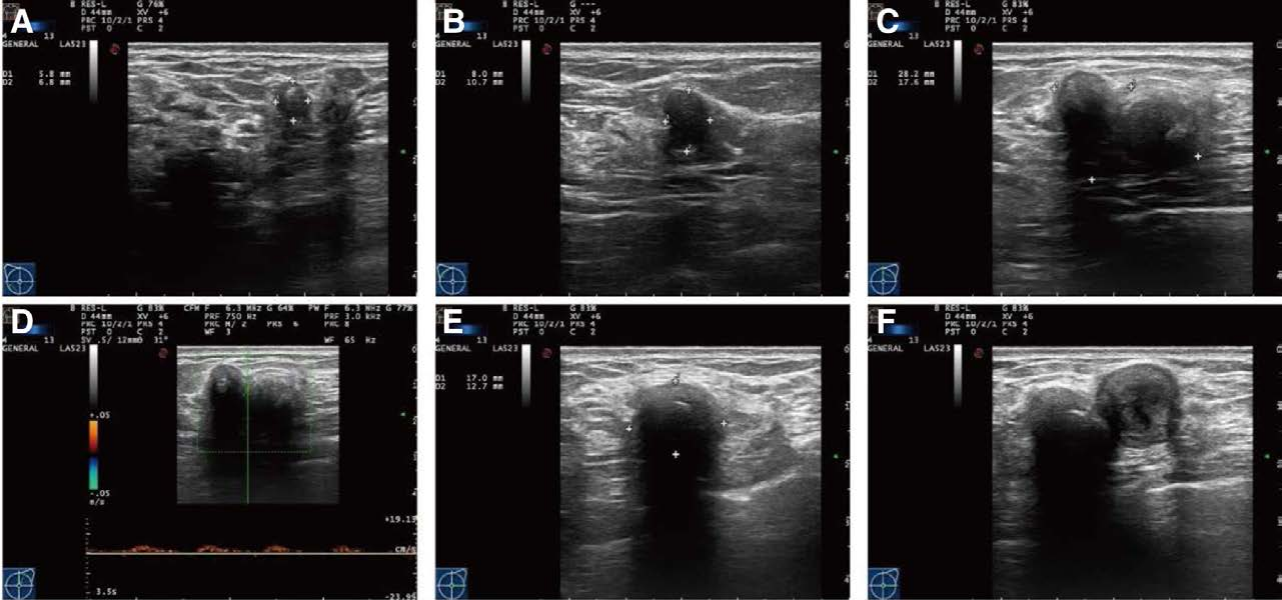
Results of bilateral breast color ultrasound examination: (A) A right breast nodule measuring 0.6 × 0.7 cm at the 8 o’clock position, 1.9 cm away from the nipple, which was straight and had a thick light spot with uneven internal echo. The nodule was assessed as BI-RADS 4B. (B) A right breast nodule measuring 0.8 × 1.1 cm at the glandular edge at 10 o’clock, which was assessed as BI-RADS 4B. (C, D) There was a 2.8 × 1.8 cm nodule at 1 o’clock in the left breast, 3.8 cm away from the nipple, with irregular shape and unclear boundary, which was assessed as BI-RADS 4C. CDFI indicated that blood flow signals could be seen in the nodule. (E, F) A 1.7 × 1.3 cm erect hypoechoic nodule was at the 3 o’clock position in the left breast with a strong echo light spot, which was assessed as BI-RADS 4B. There seemed to be another vertical nodule fusion beside it measuring 1.3 × 1.5 cm with uneven echo, which was BI-RADS 4A. CDFI = color doppler flow imaging.

The results of breast color ultrasound examination showed that there were multiple nodules in both breasts (Fig. 1). A nodule at 10 o’clock and another nodule at 8 o’clock of the right breast had thick light spots with uneven internal echoes, which were assessed as BI-RADS 4B. There was a 2.8 × 1.8 cm nodule at 1 o’clock in the left breast, with irregular shape and unclear boundary. This largest nodule in the left breast was assessed as BI-RADS 4C, and color Doppler flow imaging indicated that blood flow signals could be seen in the nodule. Another erect hypoechoic nodule was at 3 o’clock in the left breast with a strong echo light spot, which was BI-RADS 4B.

**Figure 2.**
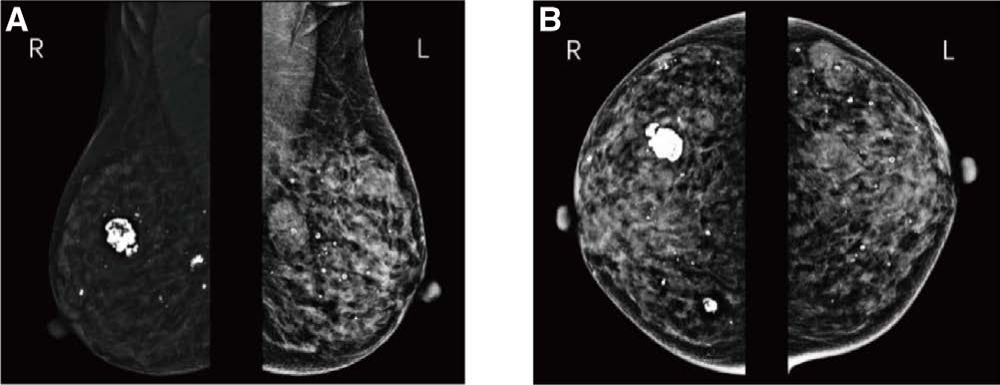
(A) Results of bilateral mammography (MLO position). (B) Results of bilateral mammography (CC position).

Mammography examination showed multiple nodules in both breasts (Fig. 2). The largest nodule in the left breast was located behind the nipple with a size of approximately 2.5 × 1.9 cm and had a clear boundary with small punctured calcification inside, which was assessed as BI-RADS 4B. The largest nodule in the right breast was located in the outer quadrate with a size of approximately 1.0 × 1.2 cm, which had an irregular shape and a clear boundary, with several punctured calcifications in both breasts. Residual nodules were all assessed as BI-RADS 4A and should be followed up.

**Figure 3.**
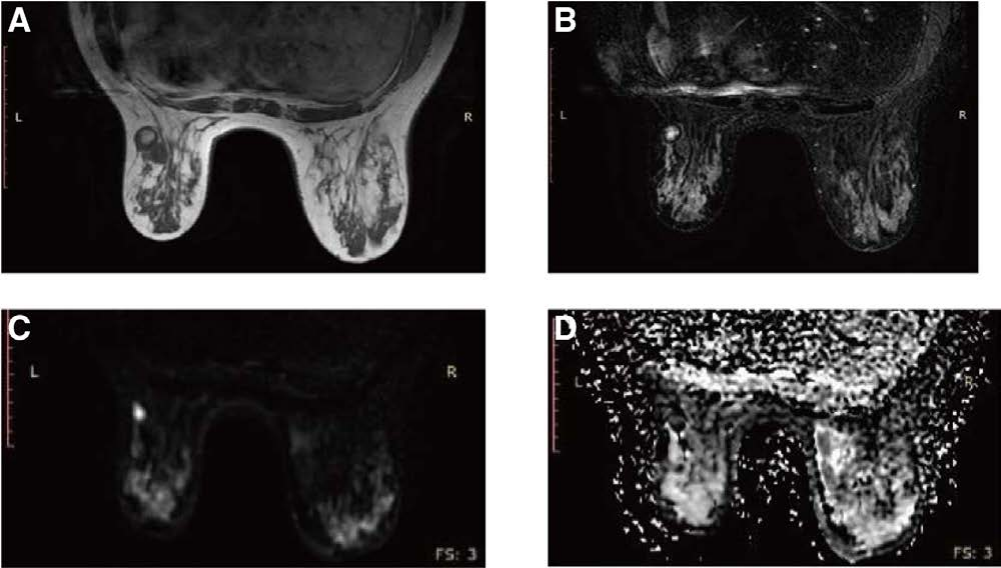
(A) T1-weighted fat saturated (T1WFS) sequence magnetic resonance imaging (MRI) of a mass in the outer lower quadrant of the left breast demonstrated a tumor with uniformly high internal signal intensity. (B) T2-weighted short-time inversion recovery (STIR) MRI demonstrated an uneven signal intensity tumor with low signal intensity of the external edge. (C, D) The tumor showed high signal intensity on diffusion weighted image (DWI) (C) and a signal cavity on the apparent diffusion coefficient (ADC) graph (D).

The results of enhanced magnetic resonance imaging (MRI) and diffusion imaging showed multiple nodules in the left breast (Fig. 3). The largest 1 measuring approximately 2.9 × 1.7 cm was located in the outer and lower quadrant, which was a long T1 mixed with T2 signal shadow, irregular shape, nodular fusion trend, shallow lobulation, partial high signal in diffusion weighted image (DWI), low signal in apparent diffusion coefficient (ADC), and obvious enhancement after enhanced scanning. The enhancement curve presented a type II time signal. The multiple nodules were all graded as BI-RADS 4A and needed needle biopsy to clear the pathology. Based on the above clinical manifestations and imaging findings, breast cancer was considered first.

**Figure 4.**
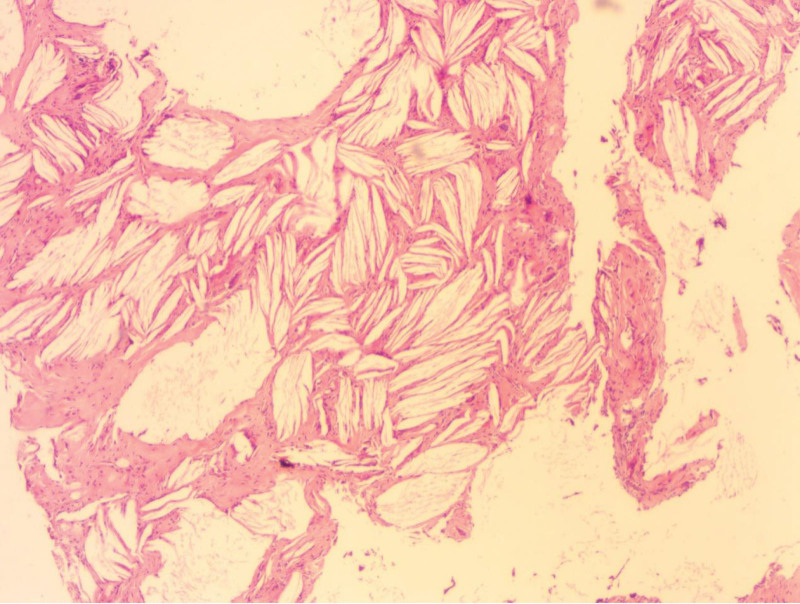
Microscopically, the cholesterol crystals were cleaved and surrounded by foreign body multinucleated giant cells. These findings were consistent with the diagnosis of cholesterol granulomas. (HE100×).

Subsequent ultrasound-guided hollow-core needle biopsy reported that all nodules were cholesterol crystals with fibrous collagen tissue hyperplasia and calcification. Due to the inconsistency between the imaging findings and the puncture biopsy results, after consultation with the patient and her family, the decision was made to perform surgical resection of local nodules in both breasts with histological biopsy. Local resection of both breasts masses was performed under general anesthesia in April 2021. The pathological results of biopsy showed that the nodules in the left breast and the nodule at 10 o’clock in the right breast were all crystals of cholesterols. The nodule at 8 o’clock in the right breast was breast adenosis with fibroadenoma formation and luminal microcalcification. The histopathological findings confirmed the diagnosis of benign breast disease: cholesterol granuloma of the breast (Fig. 4). The patient did not complain of discomfort after surgery, and ultrasound reexamination 5 months after surgery showed no recurrence.

## 3. Literature review

### 3.1. Clinical features

Multiple cholesterol granulomas of the breasts have not been reported in the published literature until now. Eleven cases of cholesterol granuloma of the breast were reviewed by us through a PubMed search of “(cholesterol granuloma) AND (breast)” (Table 1). The mean age of this patient group was 57.5 years. This is similar to the average age (57.7 years) in Gahie Nam series of retrospective studies.^[4]^ Three of these patients were found in routine physical examinations, and 2 were found after trauma or biopsy. Five patients presented with a painless and palpable hard mass that mimicked breast cancer. None of the patients reported discomfort, clinical symptoms or other signs upon physical examination. There was 1 case of breast cholesterol granuloma accompanied by cancer. The patient was 78 years old, and a movable elastic mass with a smooth surface was palpated on her right breast.^[2]^

**Table 1:** Summary of 11 patients with cholesterol granuloma of the breast.

Author	Medical record	Age (yr)	Size (cm)	Blood cholesterol level	Ultrasound	Mammography	MRI	Mammary duct ectasia	Histopathology
Takuya Osada, 2002^[6]^	routine screening	62	0.9 × 0.8	ND	irregular hypoechoic mass, not circumscribed	high-density mass, not circumscribed	ND	−	cholesterol deposition with haemorrhaging and hemosiderin
Stefania Garofalo, 2008^[7]^	one year	42	1.5	ND	irregular hypoechoic mass, not circumscribed	annular calcification shadow lesion	ND	+	cholesterol deposition with haemorrhaging and hemosiderin
Suk Jung Kim, 2019^[8]^	twenty-5 months	52	0.6–0.9	ND	progressively enlarging hypoechoic mass, no internal vascularity	not visible	ND	ND	
Young-Seon Kim, 2017^[9]^	not mentioned	48	1.4 × 0.8 × 0.7	ND	irregular hypoechoic mass, elastic hard, no internal vascularity	extremely dense breast without suspicious findings	ND	ND	cholesterol deposition with haemorrhaging and fat necrosis
Hye Shin Ahn, 2013 ^[10]^	ten years	62	5 × 4.6	ND	circumscribed, oval, complex echoic cystic mass with floating, hyperechoic internal contents	circumscribed, round, high-density mass with scattered coarse, heterogeneous calcifications present	ND	+	cholesterol deposition with haemorrhaging and fat necrosis
Masahiro Ishizaki, 2001 ^[11]^	mass was near the site of surgery 40 yr ago	74	0.8 × 0.5	ND	irregular hypoechoic mass with a coarse internal echo	IRREGULAR high-dense mass with microcalcification	ND	ND	
	routine screening	51	1.5 × 1.5	ND	irregular hypoechoic mass with a coarse internal echo	irregular high-dense mass	ND	ND	cholesterol deposition with haemorrhaging
Cheng-Chuan Hu, 2018 ^[12]^	six months of follow-up	52	0.73 × 0.53	ND	irregular hypoechoic mass with benign-looking calcifications	nodular densities with benign-looking calcifications	Homogeneous high signal intensity on T1WFS, mixed intermediate signal intensities on T2WSTIR	ND	concurrent cholesterol granuloma and invasive ductal carcinoma of the breast
Chizu furuhira, 2004^[2]^	not mentioned	78	2.0 × 1.8 × 1.3	ND	a mass with lobulated edges has 2 parts: ① a slightly hyperechoic internal echo with increased posterior echo ② a hypoechoic internal echo with decreased posterior echo	a well-circumscribed oval dense mass with smooth borders, no calcifications	ND	ND	breast cholesterol granuloma accompanied by cancer
J. Bezić, 2013^[13]^	routine screening	55	0.7	ND	hypoechoic mass with a large cyst	a large cyst	ND	ND	cholesterol deposition with haemorrhaging and macrophages
		57	0.6	ND	round hypoechoic mass with slight acoustic shadow	numerous cysts	ND	+	
This case report	two yr	61	0.6–2.8	normal	erect hypoechoic nodules with uneven internal echo and strong light spot, not circumscribed	multiple nodules, left breast masses with clear border and calcification	Obvious enhancement and Type I (benign)curve	−	breast cholesterol granuloma

+ = visible in pathological results, − = not visible in pathological results, MRI = magnetic resonance imaging, ND = not described, T1WFS = T1-weighted fat saturation, T2WSTIR = T2-weighted short-tau inversion recovery.

### 3.2. Imaging features

**Figure 5.**
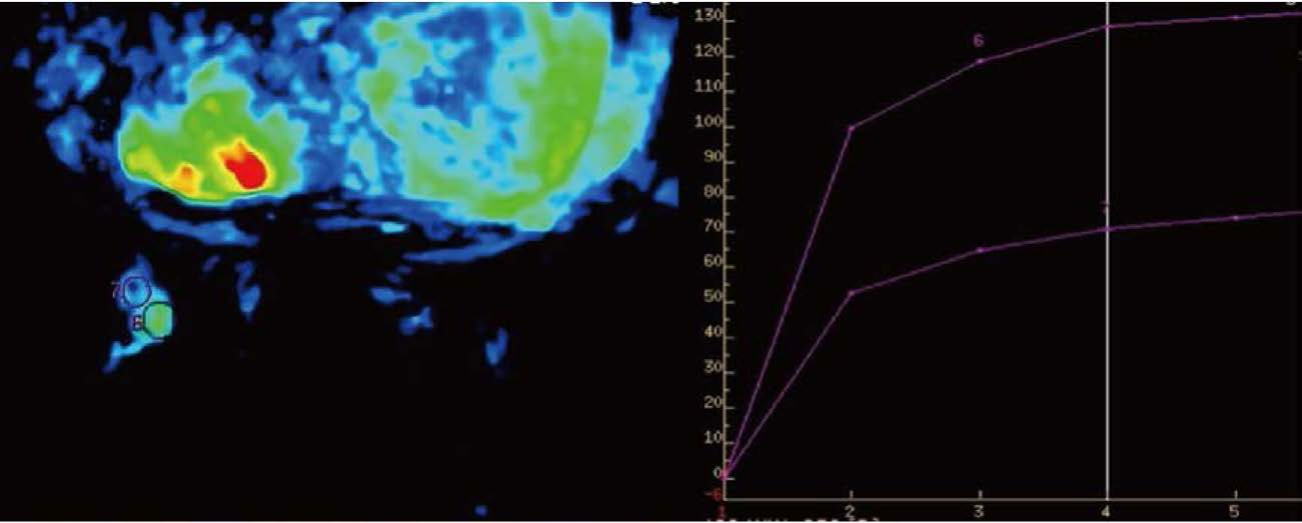
T1-weighted dynamic contrast-enhanced magnetic resonance imaging (DCE-MRI) of the left breast at the 3 o’clock position (2 fused masses) demonstrated a type II time curve.

The imaging features of breast cholesterol granulomas are not plausibly intuitive for distinguishing them from malignant breast tumors. Our collection of case reports highlighted the similarity of the mammographic and ultrasound features of cholesterol granulomas to malignant breast lesions (Table 1). As we can see, a mammogram of a cholesterol granuloma of the breast shows a high density, partial opacity and calcification. Calcification is very common and can be shown as tubular, circular and linear shadows.^[7]^ Cluster microcalcification is also present,^[12]^ which is pathologically indicated as breast cancer. The bulky, granular calcification in the case we report is unusual. Most suspicious signs on ultrasound are angular edges, unclear boundaries and irregular shapes^[7,13,14]^ with complex cystic echoes.^[11]^ It can be seen from the reviewed cases that regular, well-defined masses are mostly benign diseases. Irregular masses render further diagnosis essential. In these cases, most of the nodules had no blood flow signal. In our case, all nodules had blood flow signals. In 2 cases,^[2,12]^ the masses were composed of cholesterol granuloma and invasive carcinoma, which were difficult to distinguish on mammography and ultrasound imaging. Ultrasonography showed a heterogeneous mass composed of 2 parts.^[2]^ One had a slightly hyperechoic internal echo with increased posterior echo, and the other had a hypoechoic internal echo suggesting malignancy. Another case showed a cluster of microcalcifications classified as BI-RADS 4c on the side of an enlarging irregular hypoechoic mass. However, it could be clearly distinguished in histopathology, and septal intervals were seen distinctly between them. A case report supported the importance of using breast MRI to distinguish cholesterol granuloma from breast cancer.^[12]^ In line with our case, the cholesterol granuloma was oval, and dynamic contrast-enhanced magnetic resonance imaging (DCE-MRI) showed mild progressive enhancement as a type I time curve. The breast cancer portion showed an irregular shape, and DCE-MRI showed rapid enhancement with a Type III time curve, where the peripheral low signal edge of the mass on the T2-weighted image was most likely due to hemosiderin deposition. Diffusion limiting characteristics identified on the left breast DWI and the left breast ADC plots supported the diagnosis of cholesterol crystallization and fat deposition.^[12]^ In this case, the MRI findings of the mass in the lower quadrant of the left breast were very similar to those previously reported (Fig. 3), but the difference was that the DCE-MRI findings of the mass showed a type II time curve (Fig. 5). From these data, we can still see the critical function of breast MRI in identifying cholesterol granulomas in the breast. The appearance of the nodule in the upper quadrant of the left outer breast was regular. DCE-MRI presenting a type I time curve showed mild progressive enhancement, low signal on DWI, and empty on ADC image, which all suggested it was a benign tumor. Hemosiderin and hemorrhage were observed in multiple pathological sections of the collected cases, while low signal peripheral edges were observed on T2 images. Multiple manifestations of cholesterol granuloma on MRI have not been observed in previous case reports. In general, although cholesterol granuloma cannot be distinguished from breast cancer on mammography and ultrasound, T1-weighted DCE-MRI of the breast is apparently conducive to the diagnosis of cholesterol granuloma of the breast through its different contrast enhancement modes, diffusion limitation characteristics and time signal intensity curve.

### 3.3. Histological features

Cholesterol granuloma is a nonspecific inflammatory reaction elicited by the presence of foreign bodies, which are cholesterol crystals. This disease is relatively rare in the breast, most commonly occurring in the middle ear and mastoid process, and is also found in the parotid glands,^[15]^ lymph nodes,^[14]^ liver and spleen.^[16]^ Surgical specimens are mostly yellowish-brown masses with soft^[2]^ or solid texture.^[1]^ The section surface has a sandy texture and is clearly demarcated from normal breast tissues.^[8]^ Histologically, cholesterol granuloma is easily distinguished from other diseases. It is composed of fibrous granulation tissue with a large number of parallel or radially arranged cholesterol crystals that are deposited in the center, surrounded by foreign body giant cells and partially infiltrated lymphocytes and plasma cells.^[7]^ The walls of the expanded ducts among them are thickened and fibrous. The intima of the ducts is occasionally atrophic or incompletely gathered around with varying proportions of lymphocytes and tissue cells.^[8]^ The case of cholesterol granuloma accompanied by cancer revealed 2 components that were clearly separated macroscopically.^[2]^ One was dark brown and contained cholesterol clefts and foreign body-type giant cells. Another part was gray and hard, and histological examination revealed invasive ductal carcinoma.

### 3.4. Possible pathological mechanisms

The pathogenesis of breast cholesterol granuloma is still controversial. Two mechanisms were reported to be involved in the formation of breast cholesterol granulomas. First, the primary disease is duct dilation, which is associated with duct rupture, and lipid-rich substances escape to the periductal parenchyma, forming cholesterol crystal granulomas surrounded by a large number of foreign body inflammatory reactions.^[12,17]^ Second, periductal inflammation may be a primary lesion, leading to duct wall damage and lipid extravasation, which further leads to duct dilation and periductal fibrosis.^[10]^ Previous biopsy or breast trauma may facilitate rupture of dilated ducts, resulting in cholesterol granulomas.^[7]^ Among the cases we summarized (Table 1), 3 cases had duct dilation,^[7,13,14]^ and 1 case had a history of biopsy,^[8]^ suggesting that perhaps they were both related to cholesterol granuloma. However, in our case, the patient’s breast ultrasound and the pathological report showed no duct dilation. In addition, the patient also had no history of trauma or biopsy. A retrospective analysis suggested that cholesterol Oma was mainly found in middle-aged women and patients with elevated blood cholesterol levels.^[4]^ The formation of cholesterol crystals is presumably elicited by lipid secretion. They speculated that elevated cholesterol levels in the blood might directly or indirectly lead to increased lipid levels in breast secretions, leading to the formation of cholesteroloma. Nonetheless, the patient’s cholesterol level in our case was normal. Therefore, the connection between breast cholesterol granulomas and cholesterol levels should be further studied. Perlecan (a basal membrane TYPE CD44 protein) was produced and accumulated in the sac walls of immature granulation tissues and could capture locally oxidized low-density lipoprotein (OX-LDL).^[18]^ Then, Ox-LDL is eaten by macrophages. As a result, LDL-filled foam-like macrophages accumulate in the granulation tissue, and free cholesterol released by ruptured macrophages may crystallize locally, leading to foreign body granulomas in the cyst walls. In Case 1,^[13]^ given a large cyst that was found at the location of the granuloma, it was speculated that a cholesterol granuloma was formed after spontaneous rupture of the large breast cyst due to the above mechanism.^[13]^

### 3.5. Recommend viable diagnostic and therapeutic approaches

Cholesterol granuloma is a specific form of chronic reactive inflammation in the breast, with a prevalence of 0.54% under core needle biopsy diagnosis.^[4]^ At present, because of the malignancy of the imaging, the diagnosis of breast cholesterol granuloma is mainly based on surgical resection of the mass and histological results. Although the results of surgical resection are clear, the impact on the patient is not negligible, such as the psychological trauma and burden of patients, which even causes excessive treatment. There were cases using fine needle aspiration, which was considered to be inexpensive and well tolerated. However, due to the lack of smear materials, smears are not sufficient for diagnosis, or they can only show a few inflammatory cells and epithelial cells, which are not enough to exclude cancerous lesions and thus cannot make a definite diagnosis.^[7]^ Therefore, breast cholesterol granulomas, which constantly exhibit as masses mimicking cancer, can present a challenge for clinicians because of their poor knowledge. In fact, it is not difficult to distinguish cholesterol granuloma from breast cancer in histology with its characteristic structure. Cholesterol granulomas of the breast show parallel or radially arranged cholesterol crystals, accompanied by infiltration of foreign body giant cells and lymphocytes, which have completely different typical characteristics from breast cancer. In this case, a core needle biopsy was performed on the patient, which confirmed the presence of cholesterol clefts with calcification in the nodule of the left breast, which was considered carcinoma. The pathology finally confirmed that multiple nodules were all cholesterol granulomas. Therefore, to exclude cancerous lesions, core needle biopsy providing a tissue core for histological diagnosis may be more conducive to the diagnosis of breast cholesterol granuloma,^[7]^ thus avoiding unnecessary surgery and overtreatment.

**Figure 6.**
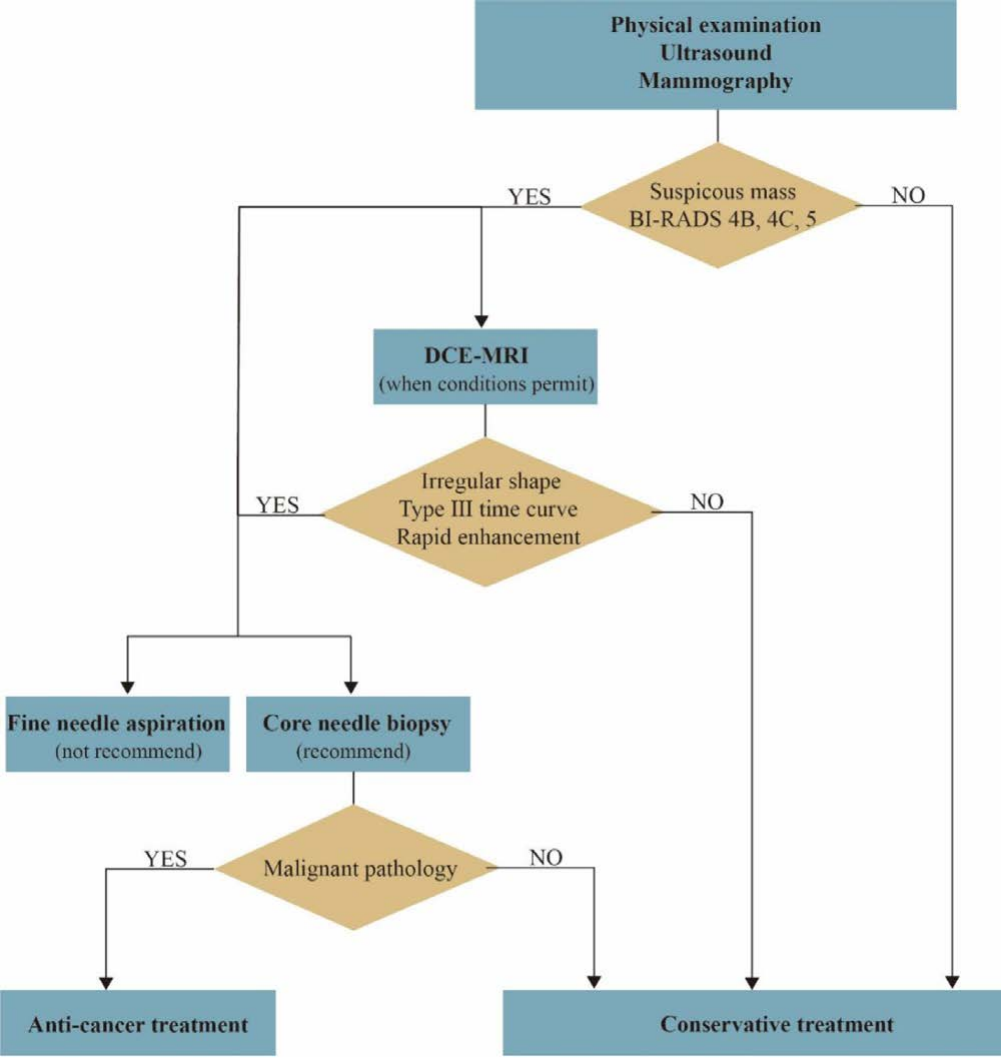
Recommend viable diagnostic and therapeutic approaches.

Nevertheless, it is a pity that patients, their families or clinicians are still considering subsequent medical burdens and treatment strategies because of the imaging reports of malignancy before the pathological results are definitely reported. In clinical practice, precision medicine pursues the correct selection and accurate application of appropriate diagnosis and treatment methods for each patient to minimize iatrogenic damage, minimize medical costs and maximize patient benefits. With advanced age, there is an increase in the accumulation of breast diseases, and accurately identifying each lesion of the breast is critical so that patients can receive the best and timely treatment. Cholesterol granuloma of the breast is a rare breast disease with ambiguous etiology that has rarely been reported but needs to be taken seriously. Importantly, the psychological trauma and burden of patients and their families being told about the different outcomes of malignant breast cancer versus benign cholesterol granuloma cannot be underestimated. To reduce excessive treatments, it is indispensable to analyze the admissible diagnosis and therapeutic approaches (Fig. 6). In regard to suspicious clinical and imaging findings, such as irregular hypoechoic nodules with unclear boundaries, at the same time, the patient’s condition and facilities permit, DCE-MRI is beneficial for diagnosis. As reported, DCE-MRI showed mild progressive enhancement as a type I time curve, while the breast cancer portion showed rapid enhancement with a type III time curve.^[12]^ The application of biopsy in clinical practice makes the diagnosis of breast diseases less intuitive and empirical from clinicians. On the 1 hand, core needle biopsy shows the most direct result. On the other hand, it is well tolerated and causes few complications. Two cases of cholesterol granulomas with microcalcification had persistent but grossly stable mass lesions on follow-up mammography after conservative treatment.^[4]^ Histopathology and follow-up advise that conservative management is appropriate in accordance with the benign nature of cholesterol granuloma. Reported in 2 cases, due to reports of cholesterol granuloma accompanied by infiltrating ductal carcinoma,^[3,19]^ surgical excision was performed after full communication with the patients and their family. Consequently, when considering the possibility of coexistence of cholesterol granuloma and cancer, surgical resection of the breast mass is recommended to confirm the diagnosis to exclude potential malignant tumors and minimize underestimation and misdiagnosis. Subsequently, we can choose the appropriate treatment based on the distinct results or advise conservative treatments for patients with cholesterol granulomas of the breast.

## 4. Conclusion

According to the clinical data and literature review of these patients, we would like to recapitulate 3 points. First, cholesterol granuloma can occur uncommonly in the breast parenchyma, and clinicians should have a good command of this benign disease. Second, the pathogenesis of breast cholesterol granuloma is still a thorny problem and needs further insight into its underlying pathomechanism. The primary lesion may be breast duct dilation or periductal inflammation, and the history of breast biopsy and trauma history may be the inducement. Middle-aged and older women with elevated blood cholesterol levels may be of particular concern.^[4]^ Most importantly, it is difficult to distinguish cholesterol granuloma from breast cancer in clinical and imaging aspects, but MRI has a certain potency in helping to distinguish cholesterol granuloma from breast cancer. In histopathology, cholesterol granulomas have a characteristic structure and can serve as the gold standard. Confirming the benign nature of cholesterol granuloma, conservative management is appropriate. Therefore, an individualized approach is essential when choosing diagnosis and treatment methods for patients with unclear imaging findings of possible malignant masses. As described above, the diagnosis of cholesterol granuloma of the breast depends on histopathological results to reduce unnecessary overtreatment and maximize patient benefits.

## Author contributions

**Conceptualization:** Qiang Wu, Bingliang Miao.

**Data curation:** Qiang Wu, Yingbo Luo.

**Formal analysis:** Chenyi Gao, Xiaoming Xu.

**Methodology:** Menglei Jin, Jing Jin.

**Supervision:** Zhigang Chen.

**Visualization:** Menglei Jin.

**Writing – original draft:** Menglei Jin, Qiang Wu, Bingliang Miao, Jing Jin.

**Writing – review & editing:** Zhigang Chen.
